# Advanced glycation end products potentiate citrated plasma-evoked oxidative and inflammatory reactions in endothelial cells by up-regulating protease-activated receptor-1 expression

**DOI:** 10.1186/1475-2840-13-60

**Published:** 2014-03-13

**Authors:** Yuji Ishibashi, Takanori Matsui, Seiji Ueda, Kei Fukami, Sho-ichi Yamagishi

**Affiliations:** 1 Department of Pathophysiology and Therapeutics of Diabetic Vascular Complications, Kurume University School of Medicine, 67 Asahi-machi, Kurume 830-0011, Japan; 2 Department of Medicine, Kurume University School of Medicine, Kurume 830-0011, Japan

**Keywords:** AGEs, RAGE, Endothelial cells, Diabetes, PAR-1, Rivaroxaban

## Abstract

Advanced glycation end products (AGEs) and receptor RAGE interaction contribute to endothelial cell damage in diabetes. Several thrombogenic abnormalities are also involved in diabetic vascular complications. However, the pathological role of thrombin and protease-activated receptor-1 (PAR-1) system in AGE-induced endothelial cell (EC) damage remains unclear. In this study, we investigated the effects of rivaroxaban, an inhibitor of factor Xa on 3% citrated human plasma-evoked reactive oxygen species (ROS) generation and RAGE, monocyte chemoattractant protein-1 (MCP-1) and intercellular adhesion molecule-1 (ICAM-1) gene expression in AGE-exposed ECs. We further examined whether FR171113, an inhibitor of PAR-1 blocked the plasma-induced EC damage and if AGEs increased PAR-1 expression in ECs. Human citrated plasma stimulated ROS generation and RAGE, MCP-1 and ICAM-1 expression in ECs, all of which were potentiated by the treatment with AGEs. Rivaroxaban or FR171113 significantly inhibited these derangements in plasma- or plasma plus AGE-exposed ECs. Moreover, AGEs significantly increased the PAR-1 levels in ECs. The present study suggests that citrated plasma could induce oxidative and inflammatory reactions in ECs via the activation of thrombin-PAR-1 system and that AGEs could potentiate the plasma-evoked EC damages via up-regulation of PAR-1. Blockade of the crosstalk between AGE-RAGE axis and coagulation system by rivaroxaban might be a novel therapeutic target for thromboembolic disorders in diabetes.

## Background

Sugars, including glucose, fructose and trioses can react non-enzymatically with the amino groups of proteins, lipids and nucleic acids to form reversible Schiff bases, and then Amadori products [1-3]. These early glycation products undergo further complex reactions such as rearrangement, dehydration and condensation to become irreversibly cross-linked, heterogeneous fluorescent derivatives called “advanced glycation end products (AGEs)” [1-3]. The formation and accumulation of AGEs in various tissues have been known to progress at a physiological aging and at an accelerated rate under hyperglycemic conditions [1-3]. There is an accumulating body of evidence that engagement of receptor for AGEs (RAGE) with the ligand AGEs elicits oxidative stress generation and resultantly evokes inflammatory and thrombogenic reactions in endothelial cells (ECs), thereby playing a central role in vascular complications in diabetes [4-9].

Several types of thrombogenic abnormalities have also contributed to the development and progression of vascular damage and thromboembolic disorders in patients with diabetes [10-13]. AGEs not only inhibit prostacyclin production in ECs through the interaction with RAGE, but also induce plasminogen activator inhibitor-1, thus promoting platelet aggregation and fibrin stabilization, thus resulting in a predisposition to thrombogenesis in diabetes [10,14]. Furthermore, the AGE-RAGE interaction stimulates tissue factor expression in ECs, being implicated in hypercoagulability in diabetes as well [15]. However, the pathological role of thrombin and protease-activated receptor-1 (PAR-1) system in AGE-induced EC damage remains unclear. Therefore, in this study, we investigated the effects of rivaroxaban, an inhibitor of factor Xa on 3% citrated human plasma-evoked reactive oxygen species (ROS) generation and RAGE, monocyte chemoattractant protein-1 (MCP-1) and intercellular adhesion molecule-1 (ICAM-1) gene expression in AGE-exposed human umbilical vein ECs (HUVECs). We further examined whether FR171113, an inhibitor of PAR-1 blocked the plasma-induced EC damage and if AGEs increased the PAR-1 expression in HUVECs.

## Methods

### Materials

An inhibitor of Xa, rivaroxaban was purchased from Tronto Research Chemicals Inc. (Toronto, Canada). Citrated human plasma from Tennessee Blood Sciences, Memphis, TN, USA. Bovine serum albumin (BSA) (essentially fatty acid free and essentially globulin free, lyophilized powder), diphenylene iodonium (DPI) and a blocker of PAR-1, FR171113 were from Sigma (St. Louis, MO, USA). D-glyceraldehyde was purchased from Nakalai Tesque (Kyoto, Japan).

### Preparation of AGE-BSA

AGE-BSA was prepared as described previously [16]. In brief, BSA (25 mg/ml) was incubated under sterile conditions with 0.1 M glyceraldehyde in 0.2 M NaPO_4_ buffer (pH 7.4) for 7 days. Then unincorporated sugars were removed by PD-10 column chromatography and dialysis against phosphate-buffered saline. Control non-glycated BSA was incubated in the same conditions except for the absence of reducing sugars. Preparations were tested for endotoxin using Endospecy ES-20S system (Seikagaku Co., Tokyo, Japan); no endotoxin was detectable.

### Cells

HUVECs obtained from Lonza Group Ltd. (Basel, Switzerland) were cultured in endothelial basal medium supplemented with 2% fetal bovine serum, 0.4% bovine brain extracts, 10 ng/ml human epidermal growth factor and 1 μg/ml hydrocortisone according to the supplier’s instructions. Rivaroxaban or AGE treatment was carried out in a medium lacking fetal bovine serum, epidermal growth factor and hydrocortisone.

### Dihydroethidium (DHE) staining

HUVECs were pre-incubated with or without the indicated concentrations of rivaroxaban for 30 min and treated with 100 μg/ml AGE-BSA, 100 μg/ml non-glycated BSA or 3% citrated human plasma in the presence or absence of 50 nM DPI, 1 mM FR171113 for 4 hr. Then the cells were incubated with phenol red free Dulbecco's Modified Eagle Medium containing 3 μM DHE (Molecular Probes Inc., Eugene, OR, USA). After 15 minutes, the cells were imaged under a laser-scanning confocal microscope. Superoxide generation was evaluated by intensity of DHE staining. The intensity was analyzed by microcomputer-assisted NIH image.

### Real-time reverse transcription-polymerase chain reactions (RT-PCR)

HUVECs were pre-incubated with or without the indicated concentrations of rivaroxaban for 30 min and treated with 100 μg/ml AGE-BSA, 100 μg/ml non-glycated BSA or 3% citrated human plasma in the presence or absence of 1 mM FR171113 for 4 hr. Then total RNA was extracted with RNAqueous-4PCR kit (Ambion Inc., Austin, TX, USA) according to the manufacturer’s instructions. Quantitative real-time RT-PCR was performed using Assay-on-Demand and TaqMan 5 fluorogenic nuclease chemistry (Applied Biosystems, Foster city, CA, USA) according to the supplier’s recommendation. IDs of primers for human RAGE, MCP-1, ICAM-1, β-actin and 18S gene were Hs00153957_m1, Hs00234140_m1, Hs00164932_m1, Hs99999903_m1, and Hs99999901_s1, respectively.

### Assay of THP-1 cell adhesion to HUVECs

Human THP-1 monocytic leukemia cells (American Type Culture Collection, Manassas, VA, USA) were maintained in RPMI 1640 medium supplemented with 1% GultaMAX (Life Technologies Corporation, Carlsbad, CA, USA) and 1% fetal bovine serum (NICHIREI BIOSCIENCES INC, Tokyo, Japan). THP-1 cells were labeled with 3 mM BCECF-AM (Dojindo, Kumamoto, Japan) at 37°C for 30 min according to the supplier’s recommendation. THP-1 cell adhesion to HUVECs was assayed according to the method described previously [17]. Briefly, HUVECs were treated with or without 100 μg/ml non-glycated BSA or 3% citrated human plasma in the presence or absence of 30 nM rivaroxaban for 4 hr, and then incubated with BCECF-AM-labeled THP-1 cells for 4 hr. After the incubation, nonadherent THP-1 cells were removed by washing the HUVECs gently. Fluorescent intensities of the adherent THP-1 cells were measured.

### Western blot analysis

HUVECs were treated with or without 100 μg/ml AGE-BSA or 100 μg/ml non-glycated BSA for 4 hr. Then proteins were extracted from HUVECs with lysis buffer, separated by SDS-PAGE and transferred to nitrocellulose membranes as described previously [18]. Membranes were probed with antibodies raised against PAR-1 (Santa Cruz Biotechnology Inc., Delaware, CA, USA) or α-tubulin (Santa Cruz Biotechnology Inc.), and then immune complexes were visualized with an enhanced chemiluminescence detection system (Amersham Bioscience, Buckinghamshire, United Kingdom).

### Statistical analysis

All values were presented as means ± standard error. Student’s *t*-test or one-way analysis of variance followed Tukey’s test was performed for statistical comparisons; p < 0.05 was considered significant. All statistical analyses were performed with the use of the PASW Statistics system (version 18.0; IBM Corporation, New York, NY, USA).

## Results

**Figure 1:**
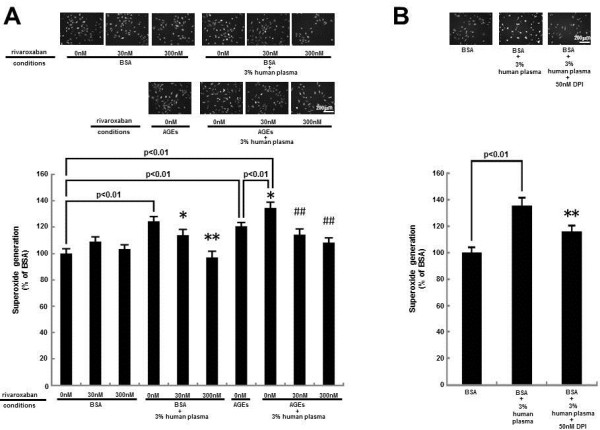
**Effects of ribaroxaban (A) and DPI (B) on superoxide generation in HUVECs.** HUVECs were pre-incubated with or without the indicated concentrations of ribaroxaban for 30 min and treated with 100 μg/ml AGE-BSA, 100 μg/ml non-glycated BSA or 3% citrated human plasma in the presence or absence of 50 nM DPI for 4 hr. Then the cells were incubated with DHE. Each upper panel shows typical microphotographs of the cells. Each lower panel shows quantitative data of ROS generation evaluated by fluorescent intensity. *N* = 5-10 per group. * and **, p < 0.05 and p < 0.01 compared to the value with BSA + 3% human plasma alone, respectively. ##, p < 0.01 compared to the value with AGEs + 3% human plasma alone.

We first examined the effects of rivaroxaban on citrated plasma-evoked superoxide generation in HUVECs. As shown in Figure 1A, rivaroxaban dose-dependently decreased the plasma-elicited ROS generation in HUVECs. Rivaroxaban itself did not affect the ROS generation in HUVECs. Furthermore, AGEs potentiated the plasma-induced ROS generation in HUVECs, which was also significantly inhibited by the treatment with rivaroxaban. An inhibitor of NADPH oxidase, DPI was found to inhibit the 3% citrated human plasma-induced ROS generation in HUVECs (Figure 1B).

**Figure 2:**
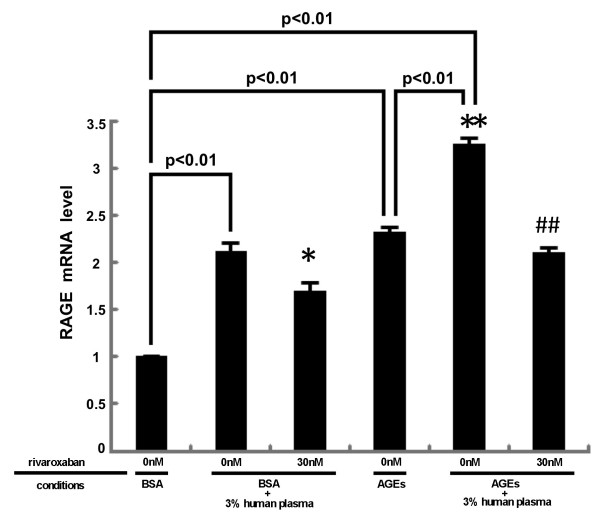
**Effects of ribaroxaban on RAGE gene expression in HUVECs.** HUVECs were pre-incubated with or without the indicated concentrations of ribaroxaban for 30 min and treated with 100 μg/ml AGE-BSA, 100 μg/ml non-glycated BSA or 3% citrated human plasma for 4 hr. Then total RNAs were transcribed and amplified by real-time PCR. Data were normalized by the intensity of β-actin mRNA-derived signals and then related to the value obtained with BSA alone. *N* = 3 per group. * and **, p < 0.05 and p < 0.01 compared to the value with BSA + 3% human plasma alone, respectively. ##, p < 0.01 compared to the value with AGEs + 3% human plasma alone.

We next studied whether plasma could up-regulate RAGE gene expression in HUVECs. As shown in Figure 2, plasma increased RAGE mRNA levels in HUVECs, which was suppressed by rivaroxaban. Moreover, AGEs further increased RAGE mRNA levels in plasma-exposed HUVEC, which was also blocked by rivaroxaban.

**Figure 3:**
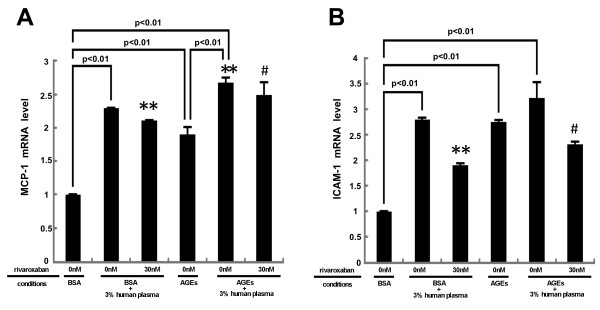
**Effects of ribaroxaban on MCP-1 (A) and ICAM-1 (B) gene expression in HUVECs.** HUVECs were pre-incubated with or without the indicated concentrations of ribaroxaban for 30 min and treated with 100 μg/ml AGE-BSA, 100 μg/ml non-glycated BSA or 3% citrated human plasma for 4 hr. Then total RNAs were transcribed and amplified by real-time PCR. Data were normalized by the intensity of 18S mRNA-derived signals and then related to the value obtained with BSA alone. *N* = 5 per group. * **, p < 0.01 compared to the value with BSA + 3% human plasma alone. #, p < 0.05 compared to the value with AGEs + 3% human plasma alone.

As the case in RAGE gene expression, rivaroxaban significantly inhibited the plasma-induced up-regulation of MCP-1 and ICAM-1 mRNA levels in HUVECs (Figure 3). These mRNA levels were further increased by the treatment with AGEs, both of which were significantly suppressed by rivaroxaban.

**Figure 4:**
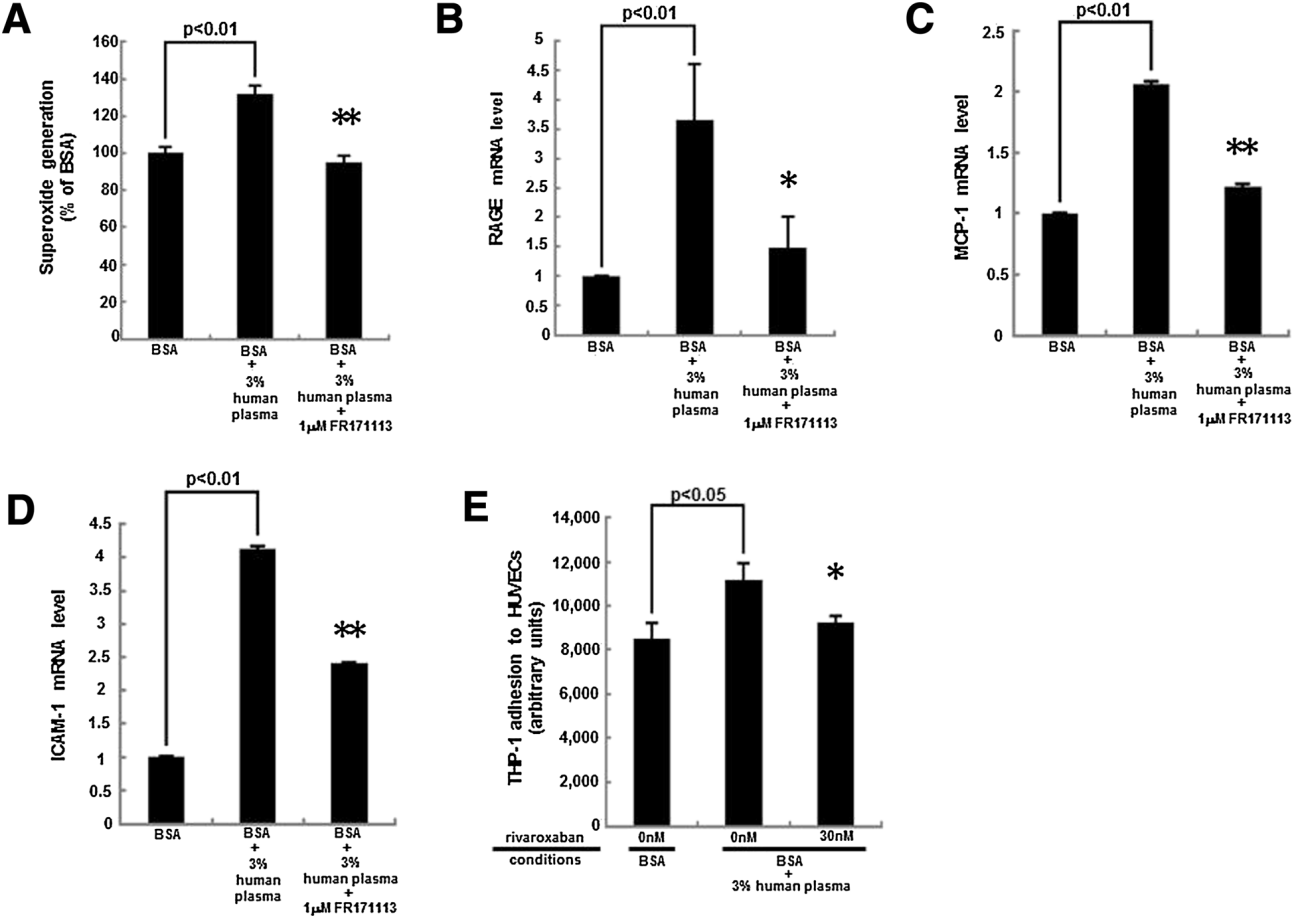
**Effects of FR171113 or rivaroxaban on superoxide generation (A), RAGE (B), MCP-1 (C), ICAM-1 (D) gene expression in, and THP-1 cell adhesion to (E), HUVECs.** HUVECs were pre-incubated with or without 30 nM ribaroxaban for 30 min and treated in the presence or absence of 3% citrated human plasma or 1 mM FR171113 for 4 hr. **(A)** Then the cells were incubated with DHE. ROS generation was evaluated by fluorescent intensity. *N* = 5 per group. **(B-D)** Total RNAs were transcribed and amplified by real-time PCR. Data were normalized by the intensity of β-actin **(B)** or 18S mRNA-derived signals **((C) and (D))** and then related to the value obtained with non-glycated BSA alone. **(E)** THP-1 cell adhesion to HUVECs was evaluated. **(B)***N* = 7 per group. **(C)** and **(D)***N* = 3 per group. **(E)***N* = 6 per group. * and **, p < 0.05 and p < 0.01 compared to the value with BSA + 3% human plasma alone, respectively.

We investigated how plasma induced the ROS generation and subsequently evoked the inflammatory reactions in HUVECs. As shown in Figure 4A, a blocker of PAR-1, FR171113 completely inhibited the plasma-induced ROS generation in HUVECs. Moreover, FR171113 significantly prevented the plasma-elicited up-regulation of RAGE, MCP-1 and ICAM-1 mRNA levels in HUVECs (Figure 4B-D). We further studied whether rivaroxaban could inhibit the plasma-evoked THP-1 cell adhesion to HUVECs. As shown in Figure 4E, 3% citrated human plasma increased THP-1 cell adhesion to HUVECs by about 1.4-fold. Thirty nM rivaroxaban significantly inhibited the THP-1 cell adhesion to plasma-exposed HUVECs.

**Figure 5:**
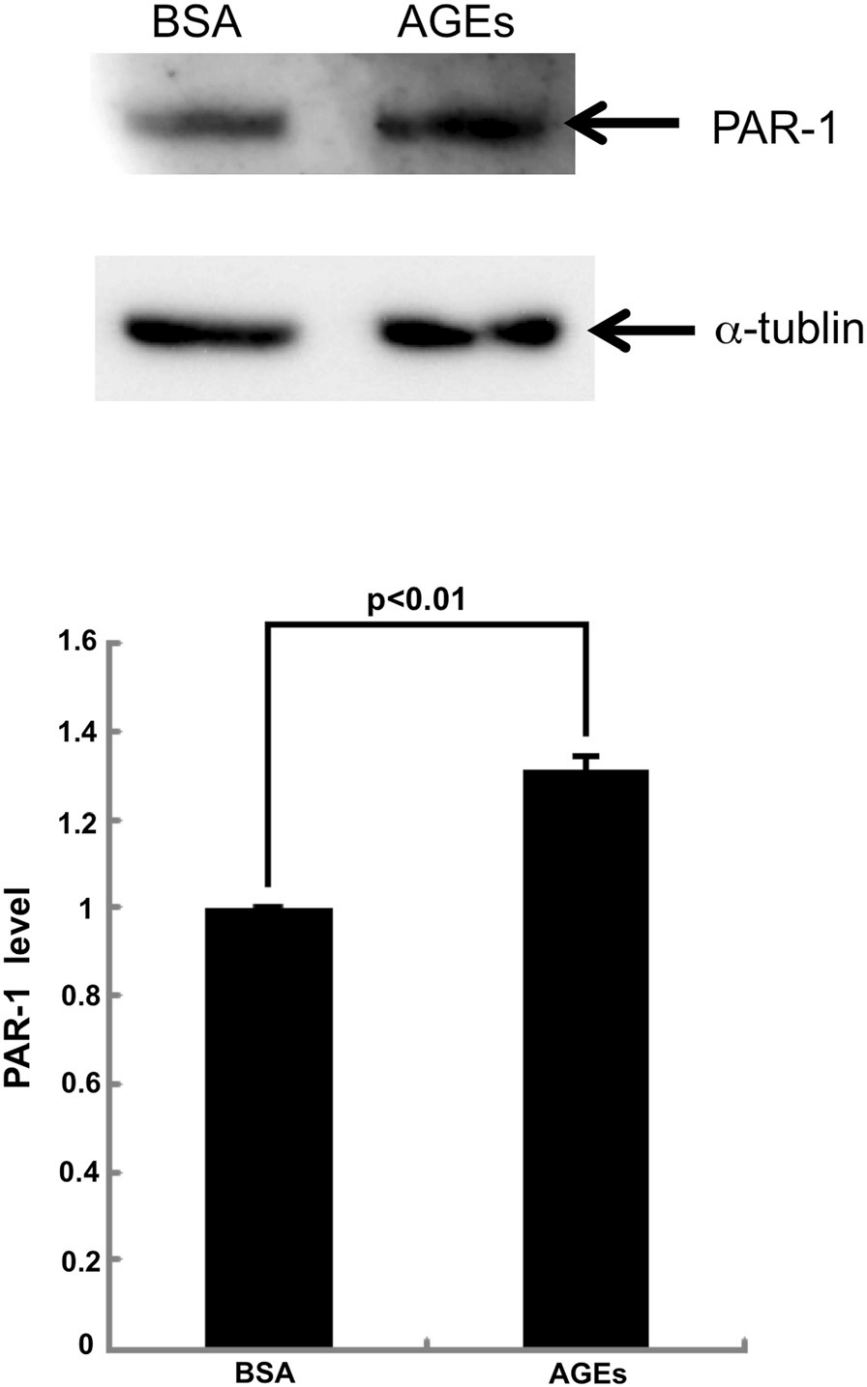
**Western blot analysis for PAR-1.** HUVECs were treated with or without 100 μg/ml AGE-BSA or 100 μg/ml non-glycated BSA for 4 hr. Then proteins were extracted from HUVECs with lysis buffer, separated by SDS-PAGE and transferred to nitrocellulose membranes. Membranes were probed with antibodies raised against PAR-1 or α-tubulin, and then immune complexes were visualized with an enhanced chemiluminescence detection system. Upper panel shows the representative bands of western blots. Lower panel shows the quantitative data. Data were normalized by the intensity of α-tubulin-derived signals and related to the value with non-glycated BSA. *N* = 3 per group.

In addition, AGEs significantly increased the PAR-1 protein levels in HUVECs; PAR-1 levels were increased to 1.3-fold of that of non-glycated BSA-treated HUVECs (Figure 5).

## Discussion

### Citrated plasma-evoked EC injury

**Figure 6:**
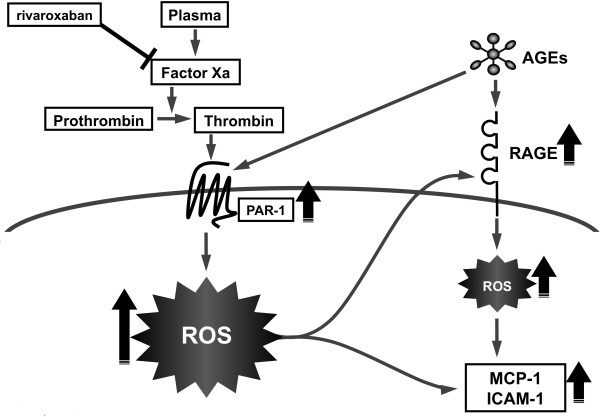
Possible participation of the AGE-RAGE axis in thrombin-evoked EC injury.

We chose the condition of 100 μg/ml AGE-BSA for 4 hr in the present experiments because we have previously found that ROS generation and inflammatory reactions of ECs reach a maximum at 4 h in the presence of 100 μg/ml AGE-BSA [16,19]. In this study, we found for the first time that 3% citrated plasma increased ROS generation, up-regulated RAGE, MCP-1 and ICAM-1 mRNA levels in, and stimulated THP-1 cell adhesion to, HUVECs, all of which were significantly blocked by the treatment with rivaroxaban, an inhibitor of Xa (Figures 1, 2, 3 and 4). Furthermore, 3% citrated human plasma-evoked ROS generation, RAGE, MCP-1 and ICAM-1 gene induction was significantly blocked by the treatment with a blocker of PAR-1, FR171113 (Figure 4). Since calcium level in HUVEC basal medium used here (6.4 mg/dl) is almost equal to that of human serum, citrated plasma is re-calcified and thrombin could be generated in the cell culture medium. Therefore, rivaroxaban could inhibit the formation of thrombin from prothrombin via blockade of Xa, thereby suppressing the thrombin-PAR-1-induced ROS generation in HUVECs (Figure 6). In the present study, we also found that an inhibitor of NADPH oxidase, DPI suppressed the plasma-induced ROS generation in HUVECs. So, re-calcified citrated plasma might evoke the ROS generation in HUVECs through he activation of NADPH oxidase via the interaction of thrombin with PAR-1. NADPH oxidase was shown to be necessary for thrombin-induced signaling pathway in vascular cells, thus supporting our speculation [20].

There is accumulating evidence that thrombin could elict the EC activation via PAR-1 [21-23]. Indeed, thrombin-PAR-1 interaction has induced MCP-1 and ICAM-1 expression in HUVECs [21-23]. We have previously shown that gene expression of MCP-1 and ICAM-1 in ECs is induced by ROS generation via the activation of nuclear factor-κB (NF-κB) [24-29]. Moreover, thrombin has been shown to induce the MCP-1 and ICAM-1 gene expression in HUVECs via NF-κB [30-33]. So, the present findings suggest that recalcified plasma may stimulate MCP-1 and ICAM-1 mRNA levels in HUVECs through transcriptional activation of redox-sensitive transcriptional factor NF-κB.

### Crosstalk between the AGE-RAGE axis and coagulation system

Risks for various thromboembolic disorders are high in diabetes [10-13]. In the present study, we found that AGEs potentiated the 3% citrated human plasma-evoked ROS generation, RAGE, MCP-1 and ICAM-1 gene expression in HUVECs (Figures 1, 2 and 3). Further, PAR-1 expression was significantly induced by the treatment with AGEs (Figure 5). These observations suggest that AGEs could enhance the plasma-evoked thrombin signaling pathways in HUVECs via enhancement of PAR-1 expression. So, rivaroxaban might block the deleterious effects of AGEs on HUVECs by reducing the thrombin formation and subsequently suppressing the thrombin-PAR-1 axis. In this study, recalcified plasma induced ROS generation and RAGE gene induction, which could further potentiate the AGE-signaling pathways in HUVECs again. Since rivaroxaban inhibited the plasma-elicited ROS generation and RAGE gene expression in HUVECs, our present study suggests that blockade of the pathological crosstalk between the AGE-RAGE axis and thrombin-PAR-1 system by rivaroxaban might be a novel therapeutic strategy for reducing the risk of thromboembolic disorders in patients with diabetes [34].

AGE-RAGE axis has been reported to mediate atrial structural remodeling in the diabetic rats, thus increasing the risk of atrial fibrillation (AF) in diabetes [35]. Furthermore, recently, both plasma levels of AGEs and soluble RAGE, a marker of RAGE activation were shown to be higher in patients with AF, positively correlated with atrial dimensions, thus indicating a pathological role for the AGE-RAGE axis in the arrhythmogenic atrial structural remodeling in humans [36]. These findings further support the clinical relevance of suppression of the AGE-RAGE axis for the prevention of AF. The peak plasma concentration of rivaroxaban after administration of single oral dose of 10 mg is reported to be about 300-400 nM [37]. So, the concentration of rivaroxaban having beneficial effects on HUVECs used in the present experiments (30-300 nM) may also be comparable to the therapeutic level which is achieved in the treatment for patients with AF.

## Limitations

In this study, *in vitro*-modified AGEs were prepared by incubating BSA with glyceraldehyde for 1 week; this process produced relatively highly-modified proteins in comparison to those *in vivo*. However, it was unlikely that extensively-modified, unphysiologic AGEs that were formed under the *in vitro*-conditions may exert non-specific effects on HUVECs because we have previously found that immunological epitope of glyceraldehyde-modified AGEs is actually present in serum of diabetic patients and that the concentration (100 μg/ml) of *in vitro*-prepared AGEs used here are comparable with those of the *in vivo* diabetic situation [38].

An *in vivo*-animal model is needed to evaluate the *in vitro*-findings of rivaroxaban observed here. Inhibition of RAGE may help to demonstrate the relation between the AGE-RAGE axis and thrombin-PAR-1 system. In addition, further clinical intervention trials are also needed to clarify whether blockade of the crosstalk between the AGE-RAGE axis and thrombin-PAR-1 axis could actually decrease the risk of thromboembolic disorders and slow the progression of atherosclerosis in patients with AF, especially in diabetic subjects. Lastly, we have to mention about the adverse effects of rivaroxaban, i.e., more bleeding, if it is widely used especially for high-risk patients.

## Conclusions

Our present observations suggest citrated plasma could induce oxidative and inflammatory reactions in ECs via the activation of thrombin-PAR-1 system, which was potentiated by AGEs. Blockade of the crosstalk between AGE-RAGE axis and coagulation system might be a novel therapeutic target for thromboembolic disorders in diabetes (Figure 6).

## Abbreviations

AGEs: Advanced glycation end products; RAGE: Receptor for AGEs; ECs: Endothelial cells; PAR-1: Protease-activated receptor-1; ROS: Reactive oxygen species; MCP-1: Monocyte chemoattractant protein-1; ICAM-1: Intercellular adhesion molecule-1; HUVECs: Human umbilical vein ECs; BSA: Bovine serum albumin; DPI: Diphenylene iodonium; DHE: Dihydroethidium; RT-PCR: Reverse transcription-polymerase chain reaction; NF-κB: Nuclear factor-κB; AF: Atrial fibrillation.

## Competing interests

This project was financially supported by Bayer AG. Dr. Yamagishi, Dr. Ueda and Dr. Fukami have received honoraria such as lecture fees from Bayer AG.

## Authors’ contributions

YI, TM, SU and KF acquired and interpreted data. SY mainly contributed to the present study, conceptualized and designed the study, acquired, analyzed, and interpreted data, and drafted the manuscript, and took responsibility for the integrity of the data and the accuracy of the data analysis. All authors read and approved the final manuscript.
